# Supplementary material for: Grass Carp Reovirus Major Outer Capsid Protein VP4 Interacts with RNA Sensor RIG-I to Suppress Interferon Response
Source: Biomolecules. 2020 Apr 6;10(4):560. doi: 10.3390/biom10040560 (PMC7226501; doi:10.3390/biom10040560)
Supplement: Supplementary file 1 [file biomolecules-10-00560-s001.zip › Figure S1.docx]

**Figure S1**


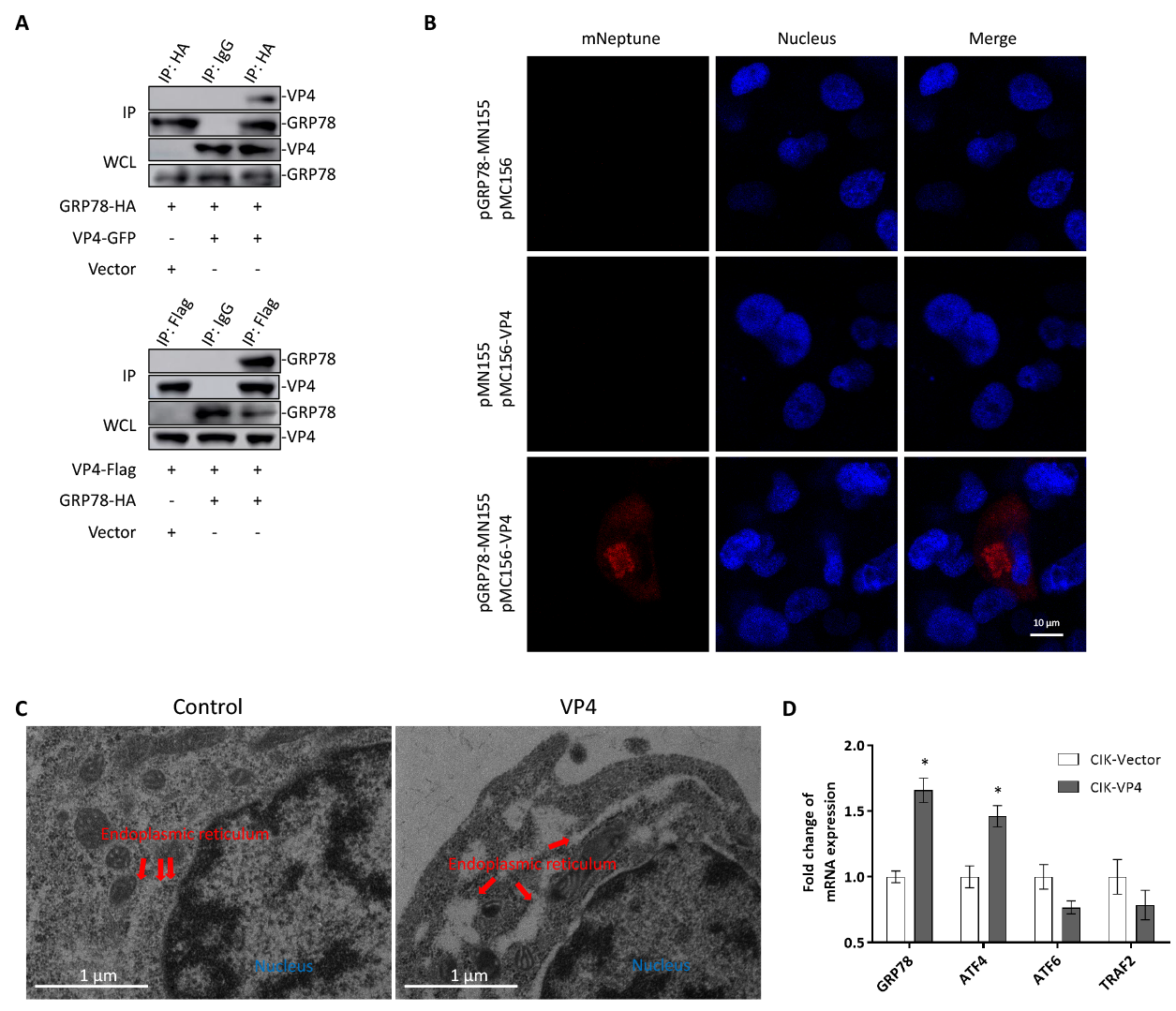


**VP4 interacts with GRP78 resulting in unfolded protein response and ER stress.** (A) VP4 interacted with GRP78. Upper: CIK cells were co-transfected with VP4-eGFP/vector and GRP78-HA for 48 h. Co-IP was performed with anti-HA monoclonal Ab and mouse IgG (control), and IB with the respective Abs. Below: CIK cells were co-transfected with GRP78-HA/vector and VP4-Flag for 48 h. Co-IP was performed with anti-Flag monoclonal Ab and mouse IgG (control) and IB with the respective Abs. (B) Imaging of the VP4-GRP78 interaction using far-red mNeptune-based BiFC *in vivo*. pGRP78-MN155 and pMC156-VP4 were transfected alone or co-transfected into CIK cells under normal conditions. In the BiFC system, the fluorescence of the mNeptune channel was red and the nucleus was stained with DAPI. The images were acquired using confocal microscopy under a 40× objective lens. Appearance of red fluorescence represents positive interaction. (C) Observation of ER deformation using TEM (transmission electron microscope). Vector/VP4 stably transfected CIK cells (CIK-vector/CIK-VP4) were fixed for 48 h and observed under a TEM. The red arrowheads indicate ER. (D) VP4 leads to ER stress via PERK-eIF2α signaling pathway. Vector/VP4 stably transfected CIK cells (CIK-vector/CIK-VP4) were seeded into 12-well plates overnight and total RNA was extracted and examined for GRP78. ATF4, ATF6 and TRAF2 mRNA expression by RT-PCR. All the co-IP, BiFC and TEM experiments were repeated independently at least three times. Data of qPCR are shown as mean ± SD of 4 wells of cell per group and are from one experiment representative of three independent experiments. Significance was calculated in relation to the control group. *P < 0.05, **P < 0.01 (two tailed Student’s tests). The relative transcription levels were normalized to the transcription level of EF1α gene and are represented as fold induction relative to the transcription level in control cells, which was set to 1.
