# Supplementary material for: Grass Carp Reovirus Major Outer Capsid Protein VP4 Interacts with RNA Sensor RIG-I to Suppress Interferon Response
Source: Biomolecules. 2020 Apr 6;10(4):560. doi: 10.3390/biom10040560 (PMC7226501; doi:10.3390/biom10040560)
Supplement: Supplementary file 1 [file biomolecules-10-00560-s001.zip › Table S1.docx]

**Table 1.** Proteins identified to potentially interact with VP4 according to GST-pull down/co-IP and LC-MS/MS analysis aligning with Uniprot-Spirulina, Uniprot-grass carp and amino acid database translated from grass carp genome and transcriptome as well as GCRV-HZ08 genome.

| UniProtKB | Gene ID | Gene name | Number of peptides | Sequence coverage |
| --- | --- | --- | --- | --- |
|  | ADJ75338.1 | VP4 | 59 | 42.15% |
| M4QQY1 |  | Retinoic acid inducible protein I | 3 | 1.06% |
| F8QPI3 |  | Retinoic acid-inducible protein I | 3 | 1.06% |
| A0A1J0CZK4 |  | Vitellogenin C | 2 | 0.89% |
| B7U616 |  | GRP78 | 2 | 4.13% |
| A0A075W4G9 |  | Kelch-like ECH-associated protein 1a | 3 | 3.13% |
| H8YJ13 |  | Mx2 | 2 | 1.59% |
| Q6TKS7 |  | Interferon-induced GTP-binding protein Mx | 2 | 1.59% |
| A0A2D0R8M9 |  | Thread biopolymer filament subunit gamma-like isoform X1 | 3 | 2.16% |
| A0A1A8A126 |  | Microtubule associated serine/threonine kinase 2 | 2 | 2.05% |
| A0A2D0R3B8 |  | Adenine DNA glycosylase isoform X3 | 2 | 2.56% |
| Q9YHX5 |  | Vimentin | 2 | 3.96% |
| W5N0K0 |  | Mitochondrial calcium uniporter regulator 1 | 2 | 3.59% |
| C9EI04 |  | Ubiquitin | 3 | 22.34% |
| A0A0F6NGK9 |  | Mitochondrial cytochrome c | 2 | 18.27% |
| A0A1S3MNK0 |  | Protein spire homolog 2 | 2 | 1.99% |
| A0A1S3MQY8 |  | Disks large-associated protein 2 isoform X2 | 2 | 1.97% |
| A0A1S3SVZ5 |  | Ankyrin repeat and KH domain-containing protein 1-like isoform X3 | 2 | 0.26% |
| B5XFR7 |  | Cytochrome c | 2 | 18.27% |
| H2M1Y0 |  | Vesicle transport through interaction with t-SNAREs 1B | 2 | 6.09% |
| W5LVL6 |  | Complement component c3b | 2 | 0.75% |
|  | CI01000082_03226861_03235247 | Vimentin | 4 | 7.27% |
|  | CI01000015_00496130_00506003 | Sentrin-specific protease 5 | 5 | 1.20% |
|  | CI01000098_01584303_01593461 | Myosin light chain kinase | 4 | 1.17% |
|  | CI01000340_02469846_02530574 | Rootletin isoform X1 | 4 | 0.31% |
|  | CI01000129_00784252_00787090 | Retinoic acid-inducible protein Ic | 3 | 3.64% |
|  | CI01000006_07731668_07752706 | Coiled-coil domain-containing protein 80 | 2 | 1.16% |
|  | CI01000010_11876103_11878638 | Protein SET | 2 | 9.17% |
|  | CI01000006_11704767_11714559 | WD repeat-containing protein 34 isoform X2 | 2 | 2.86% |
|  | CI01000050_02878198_02942065 | Vacuolar protein sorting-associated protein 13A isoform X3 | 2 | 0.50% |
|  | CI01000051_07367270_07370738 | ATP synthase subunit beta, mitochondrial | 2 | 4.96% |
|  | CI01000304_00862451_00870277 | Testis-expressed protein 15 | 2 | 0.83% |
|  | CI01000001_11019871_11028065 | Myosin light polypeptide 6-like isoform X1 | 3 | 11.24% |
|  | CI01000024_03042658_03044136 | Cytochrome c-like | 3 | 17.65% |
|  | CI01000054_12030200_12039539 | Leucine-rich repeat and WD repeat-containing protein | 3 | 1.80% |
|  | CI01000006_03893432_03940454 | Ankyrin repeat and KH domain-containing protein 1 | 2 | 0.29% |
|  | CI01000092_03365550_03416277 | Serine-protein kinase ATM isoform X3 | 2 | 0.87% |
|  | CI01180000_07100974_07142837 | Golgi-specific brefeldin A-resistance guanine nucleotide exchange factor 1 isoform X4 | 2 | 0.76% |
|  | comp65905_c1_seq15 | Vimentin | 4 | 7.28% |
|  | comp61440_c0_seq2 | Sentrin-specific protease 5 | 5 | 1.43% |
|  | comp61440_c0_seq1 | Sentrin-specific protease 5 | 5 | 1.43% |
|  | comp61638_c0_seq1 | Retinoic acid-inducible protein I | 3 | 1.06% |
|  | comp61638_c0_seq2 | Retinoic acid-inducible protein Ia | 3 | 1.06% |
|  | comp56242_c1_seq1 | Protein SET | 2 | 7.64% |
|  | comp60077_c1_seq2 | COP9 signalosome complex subunit 1 isoform X1 | 2 | 2.20% |
|  | comp64191_c0_seq1 | Testis-expressed protein 15 | 2 | 0.83% |
|  | comp64191_c0_seq3 | Testis-expressed protein 15 | 2 | 0.81% |
|  | comp65680_c4_seq1 | ATP synthase subunit beta, mitochondrial | 2 | 4.87% |
|  | comp70448_c1_seq4 | GRP78 | 2 | 4.13% |
|  | comp70448_c1_seq5 | GRP78 | 2 | 4.13% |
|  | comp70448_c1_seq7 | GRP78 | 2 | 4.13% |
|  | comp48176_c0_seq1 | Danio rerio zgc:153867 | 3 | 15.51% |
|  | comp49921_c0_seq2 | Ankyrin repeat and KH domain-containing protein 1 isoform X1 | 2 | 0.77% |
|  | comp49921_c0_seq1 | Ankyrin repeat and KH domain-containing protein 1 isoform X2 | 2 | 0.77% |
|  | comp55957_c0_seq1 | PREDICTED: PQ-loop repeat-containing protein 1-like isoform X2 | 2 | 10.98% |
|  | comp64263_c1_seq1 | G2/mitotic-specific cyclin-B3 | 2 | 3.00% |
|  | comp66718_c0_seq2 | Golgi-specific brefeldin A-resistance guanine nucleotide exchange factor 1 isoform X3 | 2 | 0.87% |
|  | comp66718_c0_seq1 | Golgi-specific brefeldin A-resistance guanine nucleotide exchange factor 1 isoform X4 | 2 | 0.87% |
|  | comp68518_c0_seq1 | Serine-protein kinase ATM isoform X3 | 2 | 0.86% |
|  | comp70388_c1_seq2 | Nesprin-1 isoform X12 | 2 | 1.11% |
|  | comp70712_c0_seq1 | Cytochrome c | 2 | 18.27% |
