# Supplementary material for: Grass Carp Reovirus Major Outer Capsid Protein VP4 Interacts with RNA Sensor RIG-I to Suppress Interferon Response
Source: Biomolecules. 2020 Apr 6;10(4):560. doi: 10.3390/biom10040560 (PMC7226501; doi:10.3390/biom10040560)
Supplement: Supplementary file 1 [file biomolecules-10-00560-s001.zip › Table S2.docx]

**Table S2**

| Sample | Raw reads | Raw bases | Clean reads | Clean bases | Error rate(%) | Q20(%) | Q30(%) | GC content(%) |
| --- | --- | --- | --- | --- | --- | --- | --- | --- |
| Control | 55687338 | 8.41E+09 | 55405104 | 8.31E+09 | 0.0233 | 98.75 | 95.79 | 47.8 |
| VP4 | 50436236 | 7.62E+09 | 50157006 | 7.52E+09 | 0.0235 | 98.68 | 95.61 | 48.4 |
| VP56 | 45321270 | 6.84E+09 | 45021564 | 6.75E+09 | 0.0235 | 98.69 | 95.66 | 47.42 |

Summary of output statistics by Illumina sequencing.
