# Supplementary material for: Grass Carp Reovirus Major Outer Capsid Protein VP4 Interacts with RNA Sensor RIG-I to Suppress Interferon Response
Source: Biomolecules. 2020 Apr 6;10(4):560. doi: 10.3390/biom10040560 (PMC7226501; doi:10.3390/biom10040560)
Supplement: Supplementary file 1 [file biomolecules-10-00560-s001.zip › Table S3.docx]

**Table S3**

Statistics of assembly quantity.

| Length | Number |
| --- | --- |
| 0~200 | 545 |
| 201~400 | 7682 |
| 401~600 | 6056 |
| 601~800 | 5095 |
| 801~1000 | 4328 |
| 1001~1200 | 3813 |
| 1201~1400 | 3255 |
| 1401~1600 | 2916 |
| 1601~1800 | 2532 |
| >1800 | 20223 |
| total | 56445 |
