# Supplementary material for: Grass Carp Reovirus Major Outer Capsid Protein VP4 Interacts with RNA Sensor RIG-I to Suppress Interferon Response
Source: Biomolecules. 2020 Apr 6;10(4):560. doi: 10.3390/biom10040560 (PMC7226501; doi:10.3390/biom10040560)
Supplement: Supplementary file 1 [file biomolecules-10-00560-s001.zip › Table S4.docx]

**Table S4**

Summary of annotation and CDS results.

|  | gene number(percent) | transcript number(percent) |
| --- | --- | --- |
| GO | 16716(0.5104) | 16716(0.5104) |
| KEGG | 17753(0.5421) | 17753(0.5421) |
| COG | 28017(0.8555) | 28017(0.8555) |
| NR | 29506(0.901) | 29506(0.901) |
| Swiss-Prot | 24426(0.7459) | 24426(0.7459) |
| Pfam | 25071(0.7656) | 25118(0.767) |
| Total_anno | 29718(0.9074) | 29765(0.9089) |
| Total | 32749(1) | 32749(1) |
