# Supplementary material for: Grass Carp Reovirus Major Outer Capsid Protein VP4 Interacts with RNA Sensor RIG-I to Suppress Interferon Response
Source: Biomolecules. 2020 Apr 6;10(4):560. doi: 10.3390/biom10040560 (PMC7226501; doi:10.3390/biom10040560)
Supplement: Supplementary file 1 [file biomolecules-10-00560-s001.zip › Table S5.docx]

**Table S5**

Primer sequences and their designated applications in the plasmid vector construction.

| Gene name | Application | Primer name | Primer sequence(5’-3’) |
| --- | --- | --- | --- |
| GRP78 | pRFP-GRP78 | GRF508  GRR509 | aattctgcagtcgacggtaccATGCGTTTCCTTTGCCTATTTT  ttatctagatccggtggatccCTAAGCGTAGTCTGGGACGTCGTATGGGTACAACTCGTCCTTTTCGCCC |
| VP4 | pGST-VP4 | VF84  VR85 | TCGCGGATCCACTATCATGGGAAACGTCCAGACG  TCTCCGCTCGAGCACGACCTAAGACGGAGGAGGCCA |
| VP4 | pVP4-Flag | VF347  VR348 | tgaaccgtcagatcgGGTACCATAGCGTGGAGACCGACTT  ggtggatccaagcttGGGCCCacCTTATCGTCGTCATCCTTGTAATCCACGACGTAAGACGGAGG |
| VP4 | pVP4-GFP | VF347  VR349 | tgaaccgtcagatcgGGTACCATAGCGTGGAGACCGACTT  ggtggatccaagcttGGGCCCacCACGACGTAAGACGGAGG |
| RIG-I | pRIG-I-MN155 | RF585  RR586 | TaggatctcgagctcaagcttATGTACGAGCTGGAAAAGGAGAA  GtaccgtcgactgcagaattcacTGAGCCGCCGCCGCCTGAGCCGCCGCCGCCTGAGCCGCCGCCGCCAGCGTAGTCTGGGACGTCGTATGGGTAGTCTCTCAGCGGCCATGTTT |
| VP4 | pMC156-VP4 | VF581  VR582 | GcatggacgagctgtacaagGGCGGCGGCGGCTCAGGCGGCGGCGGCTCAGGCGGCGGCGGCTCAATGGGAAACGTCCAGACGAA  CgcggtaccgtcgactgcagCTACTTATCGTCGTCATCCTTGTAATCAGACGGAGGAGGCCAGTATC |
| GRP78 | pGRP78-HA | GGF510  GGR511 | tgaaccgtcagatcgggtaccATGCGTTTCCTTTGCCTATTTT  AtggtggcgaccggtggatccacAGCGTAGTCTGGGACGTCGTATGGGTACAACTCGTCCTTTTCGCCC |
| GRP78 | pGRP78-MN155 | GF577  GR578 | TaggatctcgagctcaagcttATGCGTTTCCTTTGCCTATTTT  GtaccgtcgactgcagaattcacTGAGCCGCCGCCGCCTGAGCCGCCGCCGCCTGAGCCGCCGCCGCCAGCGTAGTCTGGGACGTCGTATGGGTACAACTCGTCCTTTTCGCCC |
| EF1α | qRT-PCR | EF125  ER126 | CGCCAGTGTTGCCTTCGT  CGCTCAATCTTCCATCCCTT |
| VP4 | qRT-PCR | VF146  VR147 | CGAAAACCTACCAGTGGATAATG  CCAGCTAATACGCCAACGAC |
| VP1 | qRT-PCR | VF71  VR72 | GCAATACGCCTCTACTTACTGTTCT  ATCGCTTTCTCCACCTCGTCT |
| VP56 | qRT-PCR | VF73  VR74 | AGCAGGCTATTCATCACCAGT  GTTCTAACGCTCACCGTCTTTTC |
| NS38 | qRT-PCR | VF75  VR76 | TCTGCTCCGCTTAGAAATGACTC  GACGTGGGACAATATGACAACCT |
| VP35 | qRT-PCR | VF77  VR78 | AATGTCAATTCCACCACCCC  CCTTCAGATTCACTATTCCCTCC |
| RIG-I | qRT-PCR | RF230  RR231 | ACTACACTGAACACCTGCGGAA  GCATCTTTAGTGCGGGCG |
| IPS-1 | qRT-PCR | IF217  IR218 | GACCGTAAGAAGTCAGCCTCC  CCTGAATAACTCTTGATAGCCCTC |
| STING | qRT-PCR | SF79  SR80 | TCTTATGCTGGTGTTTGCGTG  CTTTGCCTTGAATGAACGAGC |
| TBK1 | qRT-PCR | TF927  TR928 | CCAGGAGAAATGTTGGGGC  TGTAGATGTGGTGGAGTGTCGC |
| IRF3 | qRT-PCR | IF960  IR961 | ACTTCAGCAGTTTAGCATTCCC  GCAGCATCGTTCTTGTTGTCA |
| IRF7 | qRT-PCR | IF767a  IR768a | CGCCTGTGTTCGTCACTCGT  GGTGGTTGGAAAGCGTATTGG |
| IFN1 | qRT-PCR | IF590  IR591a | AAGCAACGAGTCTTTGAGCCT  GCGTCCTGGAAATGACACCT |
| IFN3 | qRT-PCR | IF435  IR357 | TACATTTATAGAGACTGCGGGTGG  TGGAGTGTCTGGTAAACAGCCTT |
| IFNγ2 | qRT-PCR | WF79  WR80 | CAGCGAACACCTGAAACTAACA  CCATCCCAAAGTCATCAAACAT |
| NF-κB1 | qRT-PCR | NF610  NR611 | CCAGGTGCGGTTTTATGAAGATGA  ATGGCTTGGGTTCGCTCGTTT |
| MyD88 | qRT-PCR | MF213  MR214 | AAAAAGGTGTAAGAGGATGGTGG  CAGGGATTAGGCGTTTAGTGC |
| TRIF | qRT-PCR | TF670  TR671 | ACCTCAGCGAGACCACCG  CTTCGTCCTAAGCACCAACG |
| GRP78 | qRT-PCR | GF81  GR82 | TGGGACGACCTACTCCTGTGTT  GCATCTCCAATGAGACGCTCT |
| ATF4 | qRT-PCR | AF83  AR84 | GCTGACTCCATCCCACATCG  GCTTAGGTGAGGGTGCCGTAT |
| ATF6 | qRT-PCR | AF85  AR86 | GCCGCACCTACAATGACTTCT  CGGCTGCCTTTGTTGTGATT |
| TRAF2 | qRT-PCR | TF87  TR88 | CGAGAAGTGTTGTCCGTGTCC  CAGGCTTCGCAGGGTATGG |
| IκBα | qRT-PCR | IF89  IR90 | TCCACGAGGCGGAAGATTAT  TCTGTGATGACGGCGAGATG |
